# An Introduction to Machine Learning for Speech-Language Pathologists: Concepts, Terminology, and Emerging Applications

**DOI:** 10.1044/2024_persp-24-00037

**Published:** 2025-04-01

**Authors:** Claire Cordella, Manuel J. Marte, Hantian Liu, Swathi Kiran

**Affiliations:** a Center for Brain Recovery, Boston University, MA; b Department of Computer Science and Faculty of Computing & Data Sciences, Boston University, MA

## Abstract

**Purpose:**

The purpose of this article is to orient both clinicians and researchers to machine learning (ML) approaches as applied to the field of speech-language pathology. We first introduce key ML concepts and terminology and proceed to feature exemplar papers of recent work utilizing ML techniques in speech-language pathology. We also discuss the limitations, cautions, and challenges to the implementation of ML and related techniques in speech-language pathology.

**Conclusions:**

Readers are introduced to broad ML concepts, including common ML tasks (e.g., classification, regression), and specific types of ML models (e.g., linear/logistic regression, random forest, support vector machines, neural networks). Key considerations for developing, evaluating, validating, and interpreting ML models are discussed. An application section reviews six exemplar published papers in the aphasiology literature that have utilized ML approaches. Lastly, limitations to the implementation of ML approaches are discussed, including issues of reliability, validity, bias, and explainability. We highlight emergent solutions and next steps to facilitate responsible and clinically meaningful use of ML approaches in speech-language pathology moving forward.

Machine learning (ML) has become an increasingly popular approach for diagnosis and prognostication in the medical and rehabilitation realms, including within the field of speech-language pathology (Adikari et al., 2023; Azevedo et al., 2023; Miller et al., 2023; Varoquaux & Cheplygina, 2022). ML refers to a branch of computer science focused on the development of models and algorithms that “learn” from data in order to improve predictive performance (Miller et al., 2023). ML is a subfield of artificial intelligence (AI) more broadly, the latter of which focuses on equipping machines with capacities to approximate humanlike intelligence (e.g., communication, reasoning, perception). The goal of ML is to have machines learn from vast amounts of data, typically to solve a circumscribed problem (Toh et al., 2019). A major advantage of ML is the ability to learn from training data and generalize to untrained or unseen data. Because of its reliance on large, heterogeneous data inputs, ML is intricately tied to the broader concept of “Big Data,” which refers to extremely large, heterogeneous collections of data that are often rapidly collected and difficult to process using traditional data handling or analysis techniques (De Mauro et al., 2015; Favaretto et al., 2020).

The emergence of ML in speech-language pathology has its roots in several concurrent trends. First is the increasing availability of Big Data that enables deployment of data-hungry ML techniques. Big Data sources include large, curated databases such as the AphasiaBank or PhonBank (MacWhinney & Fromm, 2016; Rose & MacWhinney, 2014) as well as more ad hoc data sources that have emerged as speech-language researchers increasingly employ remote monitoring approaches (Cordella et al., 2022; Kadambi et al., 2023; Liu et al., 2023) or pool years of research data on a specific population or theme (Benway et al., 2023). These approaches include wearable sensors (Cao et al., 2023; Coyle & Sejdić, 2020; Van Stan et al., 2015), smartphone digital recordings (Connaghan et al., 2019; Kadambi et al., 2023; Van Stan et al., 2017), and ecological momentary assessment (Hester et al., 2023; Marks et al., 2021) to name a few. These approaches have the advantage of capturing real-time data with considerable ecological validity but yield as a result large, noisy, and complex data sets that require prohibitive expenditures of labor and expertise to analyze manually and thus are well suited to ML analysis approaches.

A second reality of research trends in speech-language pathology that sets the stage for ML approaches is an increasing acknowledgement of the heterogeneity of patient presentations, treatment response, and recovery trajectories (Fridriksson & Hillis, 2021; Kiran, 2016; Kristinsson, Basilakos, et al., 2021; Kristinsson et al., 2022), leaving clinicians unsure of which treatment may work best for which person and in what context. Small-group studies coupled with traditional statistical or Bayesian approaches may obscure this important interindividual heterogeneity, with the result being limited generalizability of results. Although ML approaches can face the same pitfalls, their inclusion of large and diverse data sets and focus on validation techniques (e.g., cross-validation) promise increased generalizability to never-before-seen patient cases (Kernbach & Staartjes, 2022).

A final reality within the field of speech-language pathology that encourages development of ML and other automated approaches to assessment and monitoring is the documented shortage of qualified speech-language pathologists (SLPs) nationally and internationally (American Speech-Language-Hearing Association, 2022; Royal College of Speech and Language Therapists, 2023), which is especially acute in certain settings (e.g., schools) and geographic locations (e.g., rural areas; Farquharson et al., 2022; E. Kelly et al., 2023; Morton et al., 2022). Recent research also suggests disparities in referral rates and access to quality speech-language services that tend to disadvantage minoritized populations (Elliott et al., 2022; Rameau et al., 2023). Given these trends, ML approaches that could complement expert clinician judgment and/or reduce the workload for individual clinicians by reliably automating aspects of everyday practice are appealing and preferable to the current status quo of delayed access to SLPs or service provision by non-SLPs (Farquharson et al., 2022). Although far from a gold standard, ML has shown promise in other health care fields to improve diagnostic accuracy and prognostication relative to clinical gold-standard metrics (Moncada-Torres et al., 2021; Qiu et al., 2020; Trister et al., 2017), though tackling issues of bias remains difficult and incomplete (Huang et al., 2022; Obermeyer et al., 2019).

This tutorial is intended as a basic primer on ML as applied to the field of speech-language pathology. In this primer, we aim to (a) equip both clinicians and researchers with the introductory knowledge of ML that enables meaningful comprehension and discernment of emerging ML-related research and (b) provide a starting point for those interested in utilizing ML techniques as part of their own research. Toward those ends, we introduce key ML concepts and terminology—with a focus on theoretical approach and not mathematical specifics—and highlight examples of recent ML-focused work in speech-language pathology. Given the authors’ prior work in aphasia, we focus our examples on this population. Finally, we dedicate considerable attention in the final section of the primer to discuss limitations, cautions, and challenges to the implementation of ML and related techniques in speech-language pathology, including an explicit call for reliability and validity frameworks and a focus in clinical interpretability of results.

## Concepts and Terminology

### Introductory ML Terminology

At the interface between speech-language pathology and ML, practitioners are increasingly encountering terminology that may seem novel but often parallels concepts familiar within clinical practice. While we acknowledge that this is not an exhaustive lexicon, Table 1 is designed to serve as a practical starting point for SLPs venturing into the domain of ML by demystifying ML jargon through definitions and contextually relevant examples. Then, in the sections to follow, we delve deeper into more specifics regarding common tasks and model types typically employed in ML analyses.

### Differences Between ML and Traditional Statistics

In the evolving landscape of data analysis, the distinction between ML and traditional statistics becomes increasingly pertinent to highlight. Traditional statistics, grounded in the principles of inferential and descriptive methodologies, has been crucial in performing hypothesis-driven analyses, emphasizing statistical inference, probability distributions, and parametric modeling (Hahs-Vaughn & Lomax, 2020). For example, traditional statistical methods may be employed in clinical trials to determine the efficacy of new treatments based on predefined hypotheses (Berube & Hillis, 2019; Cleophas et al., 2009). In contrast, ML introduces a paradigm shift toward data-driven, algorithmic learning, characterized not only by its predictive accuracy but also by its exploratory nature. This approach leverages complex algorithms (e.g., neural networks, decision trees) that iteratively learn from data to uncover patterns and insights, as discussed in the Types of ML Models subsection.

The objectives and approaches of traditional statistics, Bayesian methods, and ML, while overlapping and complementary, diverge significantly. Traditional statistics typically begins with a predefined hypothesis, and employs models such as analysis of variance or linear regression to validate or refute these hypotheses within the constraints of statistical assumptions such as homoscedasticity or linearity (Girden, 1992; Hahn & Meeker, 1993). In contrast, Bayesian statistics takes a probabilistic approach, updating prior beliefs based on observed data to estimate prior probabilities, allowing incorporation of prior knowledge, and providing a natural framework for uncertainty quantification. Bayesian methods test hypotheses through model comparison using Bayes factors and posterior model probabilities (Gelman et al., 2013). ML, however, predominantly focuses on pattern recognition and predictive modeling, using algorithms that independently identify complex, and nonlinear relationships in data sets, often obviating the need for a priori hypotheses (Madakkatel et al., 2021; Makarious et al., 2022).

The capacity to manage data complexity also differentiates these approaches. ML techniques are well equipped at handling Big Data (Kitchin & McArdle, 2016), characterized primarily by volume, velocity, and variety, and are proficient in analyzing unstructured data, such as text, images, and speech (Jain et al., 2020). Bayesian methods offer a principled framework for incorporating prior knowledge and updating beliefs based on observed data, allowing for effective handling of complex and hierarchical models (Gelman et al., 2013). Conversely, traditional statistical methods are more effectively applied to structured data sets that have been meticulously cleaned, organized, and curated, often requiring assumptions about data distribution, exemplified by the Gaussian assumptions in linear regression models (Hahs-Vaughn & Lomax, 2020).

Next, model interpretability presents another contrast. Given their reliance on fewer parameters and clear probabilistic interpretations, traditional statistical models offer greater inherent transparency in understanding the relationships between variables. Bayesian models, while more complex, provide a natural way to quantify uncertainty and interpret results through posterior distributions and credible intervals. While emerging tools may assist in the interpretation of results, ML models are thought to sacrifice interpretability for predictive capabilities, leading to the well-known “black box” problem (von Eschenbach, 2021; Wadden, 2022), as discussed in the Interpretability, Explainability, and the “Black Box” Dilemma subsection.

Furthermore, the adaptability and flexibility inherent to ML models is unparalleled (Celik & Vanschoren, 2021). Bayesian models also offer a degree of adaptability by allowing for the updating of prior beliefs as new data become available. However, unlike traditional statistical and Bayesian models, ML models are designed to dynamically adapt to evolving data patterns. This adaptability is advantageous in fields centered around eliciting change over time, such as when monitoring treatment response over the course of therapy. For instance, ML techniques have been effectively used for accuracy prediction and classification of patient therapy behavior in mobile health systems (Bhatt et al., 2022; Shin et al., 2022). More importantly, these algorithms are also employed by mobile health systems to learn from incoming patient data, allowing for personalized treatment plans in real time, adjusting to each patient’s unique and changing needs (Dadgar-Kiani & Anantha, 2020).

In conclusion, while traditional statistics lays the groundwork for understanding and inferring relationships within data and Bayesian methods provide a framework for updating beliefs based on new evidence, ML introduces a dynamic and predictive paradigm that excels in complex, large-scale data environments. These distinctions are crucial for SLPs exploring ML, highlighting the potential these technologies can have in clinical settings.

### Tasks Suitable for ML

A task is the type of problem an ML system is developed to solve. It defines how a model should learn from input data and make a proper prediction. In the section to follow, we describe commonly used tasks for which ML is widely used. We also provide specific studies (see Table 3) as exemplar applications of these ML tasks in the context of speech-language pathology.

#### Classification

The goal of a classification task is to predict a discrete label from two or more categories based on input features. Within speech-language pathology, an example of a classification task would be the prediction of a diagnostic category (e.g., dyslexia vs. no dyslexia; healthy control vs. mild cognitive impairment vs. dementia) based on multiple input features that could either be single modality (e.g., behavioral testing) or multimodality (e.g., behavioral testing plus neuroimaging).

#### Regression

The goal of a regression task is to predict a continuous numeric value based on input features. It is used to understand the relationship between features and targets. An example of a regression task in speech-language pathology would be the predictions of a continuous, standardized test score (e.g., Western Aphasia Battery–Revised Aphasia Quotient [WAB-R AQ]) based on multiple features. Similar to classification tasks, input features can be single modality or multimodality.

#### Anomaly Detection

As the name suggests, anomaly detection can be used to detect unusual or atypical examples in a data set. In large, raw data sets, anomalies always exist, and cleaning these points is essential. While traditional statistical methods and Bayesian techniques can be used for anomaly detection, ML algorithms can often achieve superior performance, particularly in complex and noisy data sets where the volume of data makes manual examination infeasible. Anomaly detection could be applied to large data sets of speech recordings to identify atypical speech patterns or abnormalities that may warrant further investigation.

#### Clustering

Clustering is used to group similar points in the data set by finding patterns or structures from unlabeled data. In speech-language pathology, clustering can be used to, for instance, discover data-driven patterns in diagnostic data. Clustering approaches can be used to identify patient subtypes and subgroups based on criteria other than clear-cut, preexisting diagnostic labels.

### Development of an ML Model

Before introducing individual types of ML algorithms, it is beneficial to first understand how the models work and how they are developed in general. The process of model development can be split into two parts, training and testing. For a given model, the training process is meant to feed training data to that model and let it adjust internal parameters on its own. After the model converges, which means it finds optimal parameters, we can evaluate the performance of the model on the test set in what is deemed the testing process.

Model hyperparameters—as introduced in Table 1—refer to preset values used as a configuration setting to define model structure and are another important consideration for overall model development. Each algorithm can have a different number of hyperparameters, and it is necessary to experiment with different combinations of hyperparameters, execute the training and testing process on each setting, and select the optimal one for final evaluation. This process is called tuning.

When experimenting with different hyperparameters to train the model, users must monitor and minimize different types of model loss. As defined in Table 1, the loss function is used to measure the error of outputs produced by the model. The loss function can be applied to both the training set and validation set to calculate error, called training loss and validation loss, respectively. After the best model configuration is determined, users will use the test set to evaluate the final performance of the model. Note here that we need an independent validation set to select the hyperparameters rather than the test set. If the test set were to be used to compare performance of different models and/or configurations, information from the test set may influence the model—called test data leakage—and lead to overly optimistic estimates of the model’s performance.

**Figure 1.**
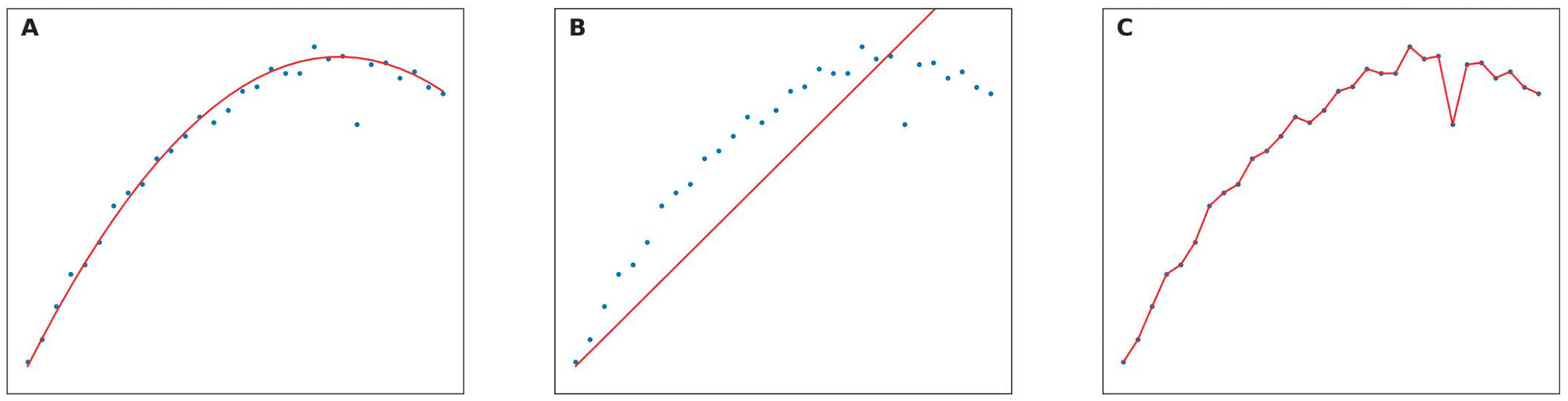
Visualization of model fit scenarios. Red curves are prediction functions generated by a machine learning model for a given data set (blue dots), demonstrating (A) good model fit, (B) model underfit, and (C) model overfit scenarios. Horizontal axis indicates a feature, and vertical axis represents the target value.

Overfitting and underfitting are two common problems that can negatively affect overall model performance. Underfitting occurs when the model fails to capture the underlying trend in the training data because of the simplicity of the model or complexity of the data set. If a model is underfit, training loss and validation loss will both be high, meaning it can predict neither seen data nor unseen data. Overfitting happens when the model is too complex, and thus, it captures not only the pattern in the data set but also in the noise and outliers. In this way, the model works perfectly on the training set, but the performance decreases markedly when unseen data are encountered. Figure 1 shows different fit scenarios for a model, including (A) a well-fit model, (B) an underfit model, and (C) an overfit model. The data are essentially a quadratic polynomial but has some noise and outliers. In Figure 1A, the model captured the quadratic nature and ignored the noise and outliers. Although it cannot predict each value precisely because of the noise, it can achieve a relatively high performance. For Figure 1B, the model produced a linear prediction because of its simplicity; thus it could not precisely predict the training set, let alone test data. On the other hand, in Figure 1C, the model perfectly predicted every sample in training set using an extremely complicated function, but it also captured the outliers, for example, the trough at the top right part of the figure. In this case, if an unseen sample follows the quadratic pattern, the error will be large.

### Types of ML Models

Having broadly introduced the tasks that are suitable to ML as well as the general process for model development, we now delve into specific types of ML models. Different types of ML models are conducive to certain tasks (e.g., regression vs. classification tasks), and ML model types can be very broadly categorized into categories of supervised and unsupervised learning. For each of these categories, we have listed common model types that one is likely to encounter in the speech-language pathology and related medical literature.

#### Supervised Learning

Supervised learning algorithms are characterized by labeled input data. In other words, these algorithm types are trained on a data set containing features, and each example is also associated with a label or target. During the training process, the model will be able to build a mapping between features and expected output. The complication for supervised ML approaches is that, in many cases, gathering labeled data can be a complex undertaking. It may, for instance, be time consuming and require expert knowledge in the field related to the data set, for example, recognizing diseased areas in medical images requires experienced radiologists to label the diseased areas in the first place in order to train the model. Some commonly used supervised learning methods are introduced below.

##### Linear regression (Suitable task: Regression).

**Figure 2.**
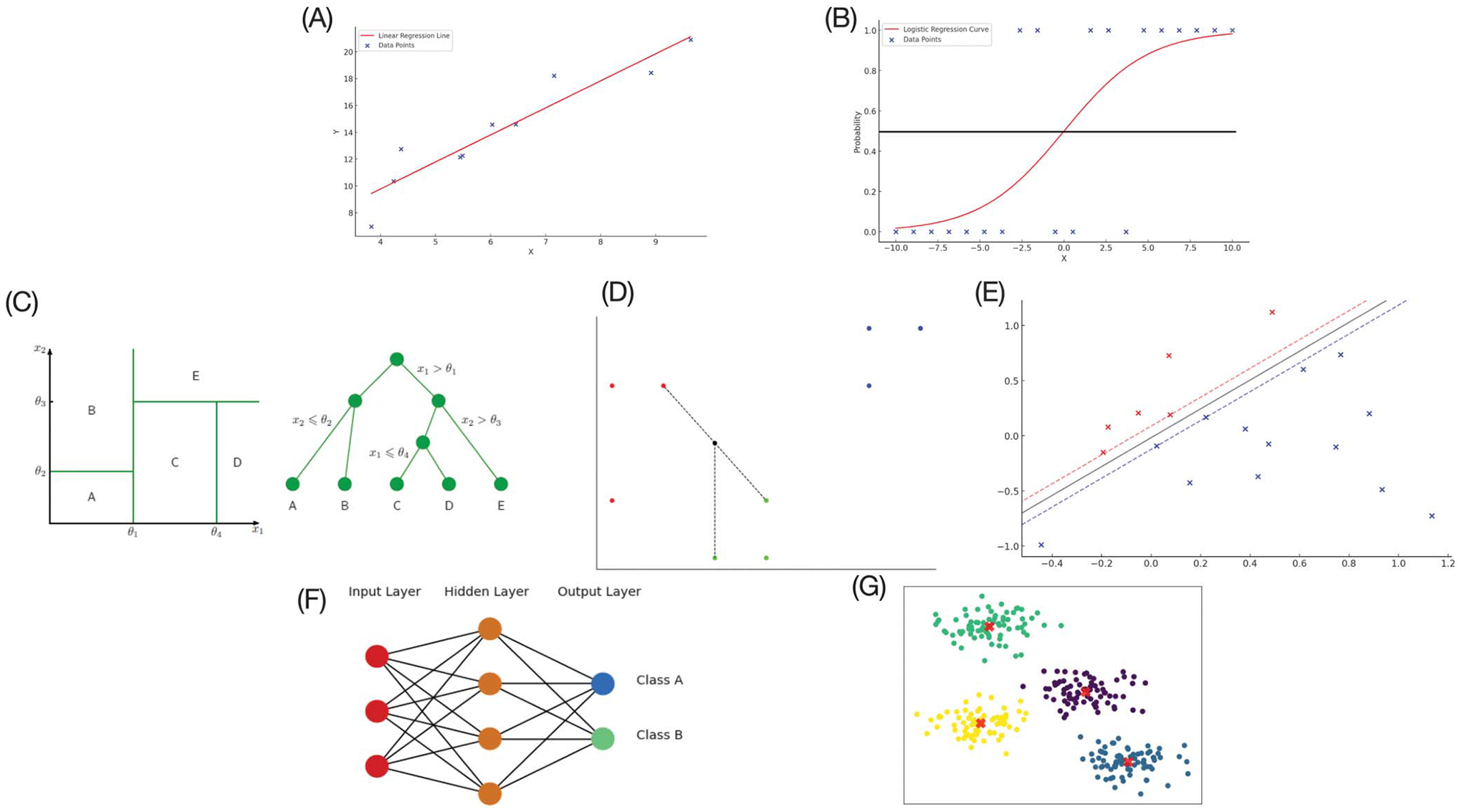
Example of different machine learning algorithms. (A) Example of linear regression. Blue crosses are data points; the red line is the prediction line learned from data. Predicted value of new data with given feature value *x* will be the corresponding *y* value on the line. (B) Example of logistic regression. Blue crosses are data points; the ones located at the top of the figure belong to the positive class, and the ones located at the bottom of the figure belong to the negative class. Red curve is the predicted probability curve, indicating the possibility of being positive at the given value of feature. Black line is 50% possibility. A new example will be predicted as positive if the possibility is more than 50% and negative otherwise. (C) Example of decision tree. Left, separated categories in feature space. Right, the decision tree generated from the pattern in left figure. From Bishop (2006, pp. 663–664), reprinted with permission. (D) Example of KNN. Points with different colors indicate different classes, and the black point is the point to be predicted. Here, three neighbors are considered. Two of them are green points, and one is red. Thus, the black point will be predicted as green. (E) Example of SVM. Black line in the middle is decision boundary, and blue and red points indicate different classes. The two dotted lines parallel to the black line pass through the closest points on each side. The algorithm can maximize the distance between the black line and each dotted line. (F) Example of neural network. In the network, there is one input layer, one hidden layer, and one output layer. (G) Example of *k*-means cluster. Initially, the data points have no class, and four cluster centers are randomly placed in the feature space. After the training process, each point belongs to the cluster whose center is closest to the point, and the position of the cluster center is the center of every point in the cluster. KNN = *k*-nearest neighbor; SVM = support vector machine.

Linear regression is one of the simplest algorithms in ML. It can discover a linear relationship between features and a numerical target value. Due to its simplicity, performance may suffer when dealing with complex data sets. Oftentimes, linear regression models are selected as a baseline model for comparison to other, more complex algorithms. Figure 2A shows an example of a prediction using linear regression.

##### Logistic regression (Suitable task: Classification).

Although the word regression appears in the name, logistic regression is an algorithm used for binary classification problems. For binary classification, labels are marked as 0 and 1, and the algorithm will predict a score between 0 and 1 for each example, representing the probability that that example belongs to Class 1. If this predicted score is less than 0.5, the example would be classified as 0; if equal to or greater than 0.5, the example would be classified as 1. Figure 2B shows an example of a prediction using logistic regression.

##### Decision tree and random forest (Suitable tasks: Classification, regression).

A decision tree is a flowchartlike tree structure used to make predictions. Each node represents a feature, and each branch represents a condition. Each example will fall into one bottom node and be assigned a value. Random forest (RF) is a set of multiple decision trees. For each example, the final result is the mode of output of all component trees. Figure 2C shows an example of a prediction using a decision tree and RF approach.

##### K-nearest neighbors (Suitable tasks: Classification, regression).

*K*-nearest neighbors (KNN) is an algorithm with a simple methodology applicable to both classification and regression tasks. Here, neighbor indicates data points close to each other in feature space, a mathematical representation where each data point is mapped to a position based on its feature values, with each feature corresponding to a dimension. Proximity in feature space indicates greater similarity between data points. For each new example, it is classified as the class most common among its KNN or predicted as their average for regression problems. Figure 2D shows an example of a KNN prediction.

##### Support vector machine (Suitable tasks: Classification, regression).

Support vector machines (SVMs) compute an *n* − 1 dimensional decision boundary on an *n*-dimensional data set. The decision boundary is an *n* − 1 dimensional hyperplane, which is a flat surface that divides the *n*-dimensional space into two parts, such as a line in a two-dimensional space and a plane in a three-dimensional space. The goal is to select a hyperplane that separates the data set into different classes while maximizing the margin, that is, the distance between the nearest points of each class (support vectors) and the hyperplane. This ensures the best possible separation between classes. In cases where the data are not linearly separable in the original *n*-dimensional space, SVMs apply a kernel function, which is a mathematical function that transforms the input data into a higher dimensional feature space without explicitly computing the coordinates in that space. In this new space, a linear decision boundary can be found that corresponds to a nonlinear decision boundary in the original space. Figure 2E shows an example of an SVM prediction in two-dimensional space, where the decision boundary is a one-dimensional line. However, SVMs can work in much higher dimensional spaces, where the decision boundary becomes a hyperplane.

##### Neural network (Suitable tasks: Classification, regression, anomaly detection, clustering).

Neural networks are one of the most powerful ML methods. This approach is inspired by the human brain, mimicking the process of signal transmission between neurons. It consists of interconnected groups of artificial neurons and can simulate complex functions. Other than the feedforward neural network shown in Figure 2F, the convolutional neural network and recurrent neural network are also used for tasks involving images or time sequences. In many cases, neural networks are used for supervised learning, but some architectures are also used in other types of learning including unsupervised learning and reinforcement learning.

#### Unsupervised Learning

Unsupervised learning algorithms are trained to detect patterns and similarities in unlabeled data sets. In other words, the algorithms are trained on a data set containing many features, which then learn useful properties of the structure of the data set. In contrast to supervised learning, unsupervised learning does not require the large amount of human work and expertise necessary to generate labeled data.

##### K-means clustering (Suitable task: Clustering).

*K*-means clustering is a popular unsupervised learning method for partitioning data into separate, nonoverlap groups or “clusters.” The number of clusters *k* is predefined, and the algorithm will automatically find the center of each cluster. Each example is assigned to the cluster to whose center it is closest. Figure 2G shows an example of unsupervised *k*-means clustering.

### Model Evaluation

The choice of evaluation metrics in ML is crucial and depends on the specific objectives and constraints of the task. Different metrics provide different perspectives on how well a model is performing, and no metric is universally best for all scenarios. For example, accuracy is an intuitive metric for classification problems, but it may not properly reflect the performance of a model when the data set is imbalanced (i.e., if a data set contains 99% positive examples and 1% negative examples, an algorithm predicting everything as positive will reach 99% accuracy, but obviously it is not a good model). Table 2 shows some common evaluative metrics for each task type.

### Model Interpretation

Model evaluation metrics, as discussed in the preceding section, provide estimates of how well a given model is performing for a specified task. These metrics do not, however, have any inherent explanatory power. That is, these metrics do not tell the end-user why the model reached a given conclusion or which features were important contributors to overall model performance. To answer these questions, specific approaches to model interpretation are needed to shed light and explain how the ML model arrived at its predictions. As is discussed in more detail in the Interpretability, Explainability, and the “Black Box” Dilemma subsection, ML models need not be a “black box” to end-users, and model interpretability approaches help with this common challenge. Model interpretability is particularly indispensable when ML is applied to speech-language pathology and other clinical disciplines, as each model needs to be meaningful to a potential clinician user of the technology (Ramanarayanan et al., 2022).

In this section, we discuss different approaches to model interpretation, including both model-specific and model-agnostic approaches. We note also that model interpretability is not simply a post hoc process but in fact begins with careful consideration of meaningful input features to the model. Without input features that have a basic clinical relevance, it is difficult to meaningfully interpret a model even with sophisticated interpretability approaches.

For simple algorithms, the model can be easily interpreted. For example, in linear regression, each feature has a weight, and the predicted value is the sum of the product of feature and weight. When the model performs poorly, it is not complicated to examine each weight and gain insights into individual feature importance in this way. Decision trees are another example of a model type that is fairly easy to interrogate. The result is produced by multiple decisions; for every sample, if one feature satisfies a rule, the sample goes to one branch, else it goes to the other branch. It is thus possible to visualize and comprehend by a human. In the KNN algorithm, it is also simple to understand the behavior of the model as it classifies the new sample as the mode of its neighbors. However, it can be hard to interpret why certain points are close to others in high-dimensional space.

**Figure 3.**
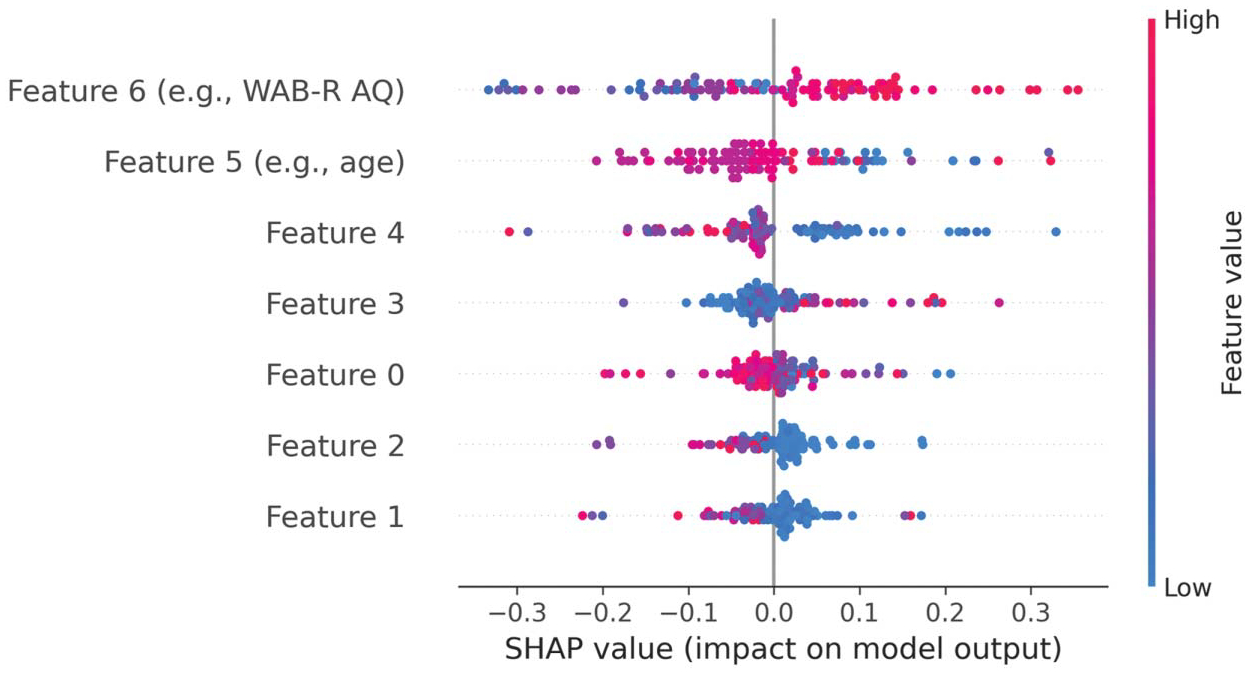
Example of a SHAP analysis feature importance plot. Each dot indicates a sample in the data set; blue dots represent lower numerical values and red dots represent higher ones. Positive value indicates positive impact on prediction. For example, in a binary classification problem (e.g., therapy responder vs. nonresponder), higher numerical value of Feature 6 (e.g., severity as indexed by the Western Aphasia Battery–Revised Aphasia Quotient [WAB-R AQ]) is associated with positive SHAP value, meaning that higher WAB-R AQ scores positively predict responder status. Feature 5 (e.g., age) shows the opposite pattern; lower scores (i.e., younger age) positively predicts responder status. SHAP = SHapley Additive exPlanation.

Other model types, especially deep neural networks (DNNs), are hard to interpret by examining the model itself, and thus alternative methods have been developed to interpret these more “black box” models. SHapley Additive exPlanation (SHAP) is one of the most widely used methods. It employs a game theoretic approach to measure the contribution of each feature to the prediction as a whole. Each feature is assigned a value based on its contribution. The larger the absolute SHAP value, the more important the feature is. Figure 3 provides a visual example of SHAP output, wherein individual features are rank-ordered from most to least important (top to bottom) based on their average absolute SHAP value. This common SHAP visual also gives information about the direction of influence of a particular feature on the model as a whole. In a hypothetical example predicting whether someone responds or not to language therapy (i.e., responder vs. nonresponder), responder status may be positively predicted by higher values for one feature (e.g., severity as indexed by WAB AQ, similar to Feature 6 in Figure 3) and lower values for another feature (e.g., age, similar to Feature 5 in Figure 3).

## Applications to Speech-Language Pathology

In this section, we will highlight exemplar papers—selected from the aphasiology literature—to orient readers to practical implementations of ML approaches that have been defined and introduced thus far. We have selected papers that utilize ML for one of two primary end goals: (a) subgroup diagnosis and/or severity characterization and (b) prediction of recovery and/or prognostication. Because our discussion is intended as an illustrative overview, we limit our focus to only a handful of papers in each section. For a more in-depth review of ML and AI approaches in aphasia, we refer readers to recently published scoping reviews by Adikari et al. (2023) and Azevedo et al. (2023). For reviews of current ML and AI trends outside of aphasia in other speech-language subdomains, we likewise refer readers to relevant recent publications by Al-Hussain et al. (2022), Minissi et al. (2022), and Zhang et al. (2023).

### Subgroup Diagnosis and Severity Characterization

As speech pathologists, correctly diagnosing a patient and estimating impairment severity are among the first and most important of clinical tasks, as it sets the stage for subsequent planning of treatment goals. Practically speaking, however, diagnostic assessment is far from trivial. For one, it requires expert integration of large amounts of multimodality information, much of which is time consuming, burdensome to patients, and/or costly to acquire. Second and perhaps most important, individual presentations are highly varied, not only with considerable heterogeneity between but also within diagnostic groups (Allison & Hustad, 2018; Ingram et al., 2020; Waring & Knight, 2013). Within the aphasia literature, this heterogeneity makes it difficult to draw clear diagnostic boundaries and categorization based on symptomology remains a heated and ever-evolving debate in both poststroke (Goodglass, 1993; Ingram et al., 2020; Wilson et al., 2023) and progressive aphasia (Gorno-Tempini et al., 2011; Ingram et al., 2020; Josephs et al., 2012; Mesulam, 2001; Tee & Gorno-Tempini, 2019). There is thus an appeal of ML-based diagnostic approaches that can take in vast amounts of multimodal data, find patterns, rank important features, and ultimately, inform clinician diagnoses and severity staging. In this subsection, we review four published articles that utilize ML approaches for the purposes of either severity characterization (Kristinsson, Zhang, et al., 2021; Pustina et al., 2017) or subgroup diagnosis (Fromm et al., 2022; Themistocleous et al., 2021). We have selected these exemplar papers because they use a diverse range of different ML techniques, including both regression (Kristinsson, Zhang, et al., 2021; Pustina et al., 2017) and classification (Fromm et al., 2022; Themistocleous et al., 2021) approaches. Three of these selected articles use a supervised learning approach (Kristinsson, Zhang, et al., 2021; Pustina et al., 2017; Themistocleous et al., 2021), while one (Fromm et al., 2022) utilizes an unsupervised approach.

Pustina et al. (2017; see Table 3 for methodological detail) were among the first to apply an ML-based technique to a longstanding challenge in the aphasia literature: prediction of aphasia symptomatology and severity from neuroimaging data (Meinzer et al., 2013; Price et al., 2017). Specifically, they investigated in a moderately large chronic poststroke cohort whether stacked multimodal predictions—combining lesion data, structural and functional connectivity—accurately predicted overall aphasia severity as well as subdomain performance (naming, repetition, auditory comprehension). The authors used a supervised RF approach to address this regression problem. To assess model accuracy, they performed correlations between predicted versus actual scores for each of the four main outcome measures and found significant correlations ranging from 0.79 (auditory comprehension) to 0.88 (overall aphasia severity). It should be noted that model accuracy was somewhat lower (0.66) for the single picture naming model that underwent full cross-validation and out-of-sample testing. Nevertheless, the authors underscore two important takeaway points from their study: (a) Neuroimaging data can accurately predict behavioral aphasia scores, and (b) Multimodal combinations of imaging data consistently outperform even the best single-modality predictors.

Kristinsson, Zhang, et al. (2021; see Table 3) echoed this basic finding in a subsequent investigation of neuroimaging predictors of aphasia severity in a very large chronic poststroke aphasia cohort. These authors utilized a supervised support vector regression technique as their main analysis model. These authors showed predictive accuracy values (i.e., correlations between actual and predicted scores) for multimodal models that ranged from 0.53 (naming) to 0.67 (overall aphasia severity). Compared to Pustina et al. (2017), Kristinsson and colleagues assessed a broader range of predicted behavioral outcomes, including overall aphasia severity, fluency, spontaneous speech, naming, repetition, and auditory comprehension. They were thus able to conclude not only that multimodal (i.e., combining data from complementary imaging modalities) models perform superiorly to single modality models but also that specific language outcomes were best predicted by different neural predictors.

Taken together, these papers exemplify potential applications of ML to predict aphasia symptomatology using supervised regression approaches (i.e., RF, SVM), with overall promising results. Though results are far from uniform and yet to be clinically validated, these types of analysis have the potential down the road to help guide clinical decision making on, for example, which neuroimaging data may be most informative to aphasia symptomatology. Note that both of these papers used regression-based ML techniques since their goals were to predict continuous behavioral outcomes. In contrast, ML approaches can also be used for classification problems, which, in the aphasiology literature, often takes the form of subgroup diagnosis. Below, we review two articles utilizing ML for this purpose, although they differ considerably in their specific approach, with one (Themistocleous et al., 2021) using a supervised approach to classification and the other (Fromm et al., 2022) using an unsupervised classification approach.

Themistocleous et al. (2021; see Table 3) used a supervised classification approach to determine whether acoustic and linguistic features of connected speech could be used to accurately predict primary progressive aphasia (PPA) subvariants. The authors compared four different types of ML approaches, namely, DNN, RF, SVM, and decision trees. Direct comparison of a comprehensive suite of different ML models is increasingly common (e.g., see Halai et al., 2020; Matias-Guiu et al., 2022), and results across these studies illustrate that the best performing model type is quite dependent on the specific research question. In other words, there is no one ML model type that is universally “best.” Themistocleous et al. found a DNN model to have the best overall classification accuracy, with 80% of individuals correctly classified. The model was significantly more accurate in identifying cases of logopenic variant PPA (lvPPA; 95% accurate) and nonfluent variant PPA (90% accurate) than it was predicting semantic variant PPA (svPPA; 65% accurate). In the case of svPPA, all misclassified individuals were erroneously identified by the model as lvPPA. Helpfully, Themistocleous et al. also include a clinical comparison to the ML models. Specifically, they had three SLPs unfamiliar with the study patients listen to the recorded speech narratives (i.e., the same data from which model input features were derived) and assign individuals into categories. The mean classification accuracy of the three naïve SLPs was lower (67%, range: 56%–78%) than the best performing ML model, meaning the model was able to predict true variant class with greater accuracy than trained SLPs exposed to the same input data as the model. This type of comparison is a first step to thinking about ways in which ML-based approaches can augment clinicians’ judgments in contexts where they are shown to have matched or superior performance accuracy; conversely, it also shows the cases (e.g., distinguishing lvPPA from svPPA) where clinicians may not wish to rely on models due to inadequate accuracy.

Fromm et al. (2022; see Table 3) utilized an unsupervised classification approach to characterize naturally occurring diagnostic clusters of patients with poststroke aphasia, using input data from connected speech samples. This unsupervised approach has the advantage of not presupposing prior patient diagnoses regarding aphasia subtype, fluency status, or the like, many of which have been called into question for their reliability and/or utility (e.g., Wilson et al., 2023). Fromm et al. specifically employed a *k*-means clustering approach, with post hoc auditory perceptual characterization intended to give clinical meaning to the data-driven clusters. Results revealed seven distinct and perceptually coherent patient clusters; these naturally occurring clusters additionally diverged in important ways from established WAB-R subtypes, illustrating the potential added value of the authors’ data-driven approach. In a unique feature of the study, Fromm et al. performed a nested classification analysis using a supervised technique (RF), in which the output of the *k*-means clustering algorithm served as the ground-truth diagnostic label for the RF analysis. The purpose of this nested analysis was to (a) compare tree grouping results to *k*-means clustering results (agreement was high at 91%) and (b) elucidate which of the 221 input variables were most important to determining the data-driven groupings. This latter analysis revealed that the groupings were largely determined by two highly salient and relatively simple variables: total number of words from a free speech task and total number of closed class words from a story retell task. This subanalysis done by Fromm et al. is an important illustration of ways to enhance ML model interpretability, as it allows readers to determine for themselves the degree to which the clustering algorithm is relying on input features that are clinically meaningful as well as the degree to which the important features in the model align with prior literature. In this example, feature importance ranking may also be a helpful cue to clinicians evaluating connected speech in the clinic, providing useful information about which assessment tasks ought to be prioritized as well as which features to pay particular attention to (i.e., two simple and easy to calculate metrics are potentially more important than more involved, labor-intensive metrics and calculations). ML results may thus be useful to clinicians not only in terms of the actual models themselves but also in terms of the mechanistic understanding they can potentially bring.

### Prediction of Recovery and Prognostication

In addition to a cross-sectional assessment of a new patient, an SLP is tasked with the subsequent development of personalized therapy goals, therapy delivery, and tracking of patient progress toward these goals. Tracking of patient progress toward functional goals gives patients and their clinicians important information about recovery, improvement or—in the case of degenerative or remittent conditions—status monitoring and prognostication. Across all subfields of speech-language pathology, there exists robust evidence that behavioral speech therapy induces recovery and/or improvements for patients (Brady et al., 2012, 2016; Law et al., 2003; Sand et al., 2022; Speyer et al., 2010); however, it is often unclear for any given patient which of several candidate therapies might be most beneficial, what the magnitude of benefit (if any) might be, or what individual patient factors might enhance/inhibit the effectiveness of therapy. These unknowns are major barriers for effective personalized speech-language therapy and offer an opportunity for ML approaches to enhance our ability to predict recovery and/or disease trajectory at the level of the individual patient.

Within aphasiology, the above conundrum of heterogeneous treatment responses has been well articulated in prior literature (Kiran, 2016; Kristinsson et al., 2022, 2023), with some authors noting the “notorious and largely unexplained variability in language recovery at the level of the individual” (Kristinsson et al., 2023, p. 1069). In other words, it has yet to be demonstrated whether predictors of response and recovery we know to be important at the group level—for instance, lesion characteristics, aphasia severity—are similarly useful in predicting individual-level responses. In recent years, a handful of investigations on this topic have emerged, with a majority of these employing various types of ML and related algorithmic approaches. In this subsection, we review two published articles that utilize ML approaches to optimize predictions of recovery following behavioral speech-language therapy (Billot et al., 2022; Grasemann et al., 2021).

In one such example, Billot et al. (2022) compare model performance of SVM and RF models for predicting responders versus nonresponders to speech-language therapy using a comprehensive multimodal feature set (neuroimaging, behavioral, and demographic data) as model inputs. The same author group published several precursor analyses with select methodological differences (e.g., see Gu et al., 2020; Lai et al., 2021) prior to the 2022 study. Results from that study demonstrated the superiority of a multimodal feature set compared to any single-feature input but also compared to the all-feature input. The latter point is a particularly important one, especially from a clinical point of view, as it illustrates the potential for ML approaches to down-select important features, that is, to identify and prioritize the most informative variables from a larger set, effectively reducing the dimensionality of the data. In the Billot et al. study, the most consistently important features to predict individual responsiveness to therapy were functional connectivity at rest, anatomical integrity, and aphasia severity. If such results are adequately replicated, it could ultimately inform which types of data clinicians should pay most attention to when thinking about therapy recommendations and approaches for new patients.

Another set of pioneering studies come from the bilingual aphasia literature, wherein prediction of language recovery is uniquely complicated by dual-language processes and the challenge of identifying the optimal language for treatment. To answer this question, Grasemann et al. (2021) developed a novel neural network, BiLex, based on self-organizing maps (SOMs). SOMs are trained via unsupervised learning algorithms, with the goal to generate a low-dimensional representation of a high-dimensional feature input space. In the BiLex model, there are three SOMs representing a shared (i.e., dual language) semantic map and two separate phonetic maps for each of the patient’s languages. The BiLex model then optimizes a set of global training parameters, combined with individual training parameters reflective of each patient’s language learning history and life span. The individual parameters, derived from self-reported language use data, adjust associative connections between the semantic and phonetic maps using Hebbian learning, a process where connections between simultaneously active neurons are strengthened, given a set of training epochs (i.e., simulated aging). Meanwhile, global parameters include learning rates, neighborhood sizes, and other factors affecting the model’s training across all individuals. In the Grasemann et al. study, the authors relied on clinical trial data from 13 Spanish–English patients with aphasia who had undergone semantic feature-based naming treatment to validate the model architecture. The goal of the study was to use BiLex to simulate prestroke naming ability and poststroke naming impairment in both of patients’ languages and predict treatment response in the treated and untreated language. Study results indicated strong potential for the computational models to predict treatment outcomes in the treated language after four therapy sessions, with coefficients of determination (i.e., actual vs. predicted values) ranging from 0.56 to 0.85. The model was less accurate, however, in predicting response in the untreated language (maximum coefficient of determination = 0.6) and tended to underestimate the degree of cross-language generalization. The BiLex computational model was extended more recently in the preliminary work of Fidelman et al. (2022) that simulated specific impairments in two individuals with svPPA. Results showed that two of the six candidate BiLex lesions fit the patient data well and that these two best fit lesions were both characterized by progressive deletion of semantic map neurons. The latter insight is another illustration of the ways in which computational models such as BiLex can potentially provide mechanistic insights into certain impairments or disorders. Taken together, this line of inquiry holds promise for clinical applications down the line that could aid SLPs in, for instance, choosing an optimal language for therapy for a bilingual patient or providing personalized prognostications of progression for individuals with degenerative conditions.

## Limitations and Challenges to ML Implementation

As the integration of ML into speech-language pathology advances, addressing the inherent limitations and challenges of these technologies becomes imperative. These challenges, encompassing aspects of reliability, validity, bias, interpretability, and the necessity for specialized training and expertise, significantly influence the effectiveness and ethical application of ML in clinical settings.

### Reliability, Validity, and Bias

ML models in speech-language pathology must consistently perform across different data sets (reliability) and make accurate predictions in clinical settings (validity). However, the nature of clinical data presents several challenges. For example, Sun et al. (2022) found that ML models for predicting heart failure events had lower clinical feasibility and reliability compared to traditional statistical models. This was largely due to the inclusion of too many predictors without careful feature selection, leading to overfitting and reduced generalizability. Similarly, an overparameterized ML model designed to predict post-stroke aphasia recovery may also suffer the same issue, fitting the noise in the training data too closely, causing its predictive performance to deteriorate when applied to new clinical populations. To address these issues, ML models in speech-language pathology must be developed using clinically relevant features and appropriate dimensionality reduction techniques and must be rigorously validated on independent data sets representative of the target population.

Additionally, Finlayson et al. (2021) highlight the challenge of data set shifts in ML models used in clinical settings. Data set shifts occur when the data used to train an ML model differ significantly from the data encountered during its actual use. These shifts can arise from variations in patient demographics, disease prevalence, or clinical practices between the training phase and real-world application. For instance, an ML model trained to predict poststroke aphasia recovery using data from a specific geographic region may not generalize well to patients from other regions due to variations in demographics, health care access, and rehabilitation practices. Subbaswamy et al. (2019) emphasize the importance of recognizing and addressing data set shifts in health care AI models, as neglecting these shifts can result in poor generalization.

Imbalanced data are another challenge, where disproportionate representation of classes in data sets can lead to biased ML models and inaccurate predictions. This imbalance often manifests in clinical data sets with a higher prevalence of common diseases compared to rare conditions. In speech-language pathology, this could translate to an ML model being trained on a data set with a higher proportion of individuals with frequently occurring communication disorders, such as articulation disorders, compared to less common conditions such as childhood apraxia of speech. To counter this, researchers can make use of specialized sampling techniques to achieve a better sensitivity–specificity balance in model predictions (see, e.g., Chawla et al., 2002; He et al., 2008).

### Interpretability, Explainability, and the “Black Box” Dilemma

The “black box” problem in ML refers to the opaqueness of complex ML models, where the internal decision-making processes are not transparent or easily understood. This issue is especially pertinent in clinical settings, where understanding the rationale behind model predictions is crucial for informed decision making. Sendak et al. (2020) emphasize the critical need for greater transparency in ML models used in health care, highlighting the challenge this poses to clinicians who must have sufficient trust in these tools prior to their use. For example, Sepsis Watch, an ML tool using diverse data to guide sepsis treatment, faced significant challenges in clinician acceptance due to its opaque decision-making process. To address this, solutions such as comprehensive staff training and stakeholder collaboration are essential for successful integration of such technologies into clinical practice (Sendak et al., 2020).

Accordingly, advancements in explainable ML have also been used to address this challenge. The essence of SHAP, as previously discussed in the Model Interpretation section, lies in its practical application for enhancing model transparency in clinical settings. For instance, SHAP can provide a relative ranking of which characteristics (i.e., features) most significantly impact the likelihood of response to treatment for a given patient, including the age of the patient, their baseline aphasia severity score, or various imaging-derived statistics (e.g., the structural integrity of specific white matter tracts), thereby guiding clinicians in personalized treatment planning.

Despite these advancements, the challenge of interpretability and explainability remains a significant hurdle in the widespread adoption of ML in clinical settings. The ongoing development of explainable ML approaches is crucial for bridging this gap, ensuring that ML models are not only effective but also transparent and understandable.

### Training and Expertise Necessary for Implementation

The effective implementation of ML in specialized fields such as speech-language pathology necessitates a deep understanding of both the clinical domain and the intricacies of ML technologies. This dual requirement presents a significant barrier to widespread adoption, as it calls for specialized knowledge and training that many speech-language professionals may not possess. Concerns around data protection, privacy, the management of health care data systems, and the ethics of model deployment further compound this challenge (C. J. Kelly et al., 2019).

For example, in radiology, ML integration necessitates an understanding of ML principles and limitations by clinicians (Grote & Berens, 2022; C. J. Kelly et al., 2019), paralleling the advancing needs in speech-language pathology. Studies in Table 3 demonstrate ML’s utility in aphasia subgroup diagnosis and outcome prediction; however, SLPs should recognize these models’ limitations, particularly in data quality and range, which may not capture the full complexity of individual aphasia cases. Likewise, Table 4 illustrates ML’s prognostic potential in aphasia treatment response. However, the probabilistic nature of these predictions and the models’ reliance on specific data sets underscore the need for cautious interpretation and integration with clinical expertise in SLP practice.

Building on this need for integration, interdisciplinary collaboration among clinicians, data scientists, and information technology professionals is increasingly vital for enhancing clinical ML systems and effectively bridging the gap between ML technology and practical health care applications (Feng et al., 2022; Sendak et al., 2020). Complementing this collaborative approach, the development of intuitive, user-friendly ML tools with graphical interfaces further reduces the need for highly specialized knowledge, making ML more accessible and practical for health care professionals (Christopoulou, 2022; Helman et al., 2022).

Finally, ethical considerations in ML extend beyond technical aspects to include societal impacts (Jo & Gebru, 2020; Prabhakaran et al., 2022). In the context of speech-language pathology, this may require clinicians being reassured that the tools and models they are using do not perpetuate biases against minority groups, which is of particular importance given how closely language is tied to cultural identity. For example, speech recognition models trained on data from majority language speakers may fail to accurately recognize speech patterns of bilingual or nonnative speakers (Bommasani et al., 2022), leading to misdiagnoses or inadequate treatment recommendations. Addressing these biases requires not only diverse data sets, including a wide range of dialects and speech patterns, but also the involvement of diverse stakeholders in the development process, ensuring that the models are inclusive and representative of all patient groups (Benway & Preston, 2023).

In conclusion, while ML/AI offer transformative potential in speech-language pathology, overcoming these challenges is crucial for their successful and ethical integration into clinical practice. The ongoing development of innovative solutions and collaborative efforts across disciplines is essential to navigate these challenges effectively.

## Conclusions

ML and other AI technologies are being rapidly developed within the field of speech-language pathology, and it is likely that such technological advancements will substantially change the way in which we both research and practice speech-language pathology. In research, ML tools offer novel ways of analyzing and finding diagnostically meaningful patterns in large and complex data sets that are not well suited to traditional statistical analysis or Bayesian methods. We have discussed several such emergent applications to speech-language pathology in this introductory primer, including prediction of poststroke aphasia severity (Kristinsson, Zhang, et al., 2021; Pustina et al., 2017), supervised subvariant diagnosis in PPA (Themistocleous et al., 2021), unsupervised cluster-based grouping of patients with poststroke aphasia (Fromm et al., 2022), and prediction of response to language therapy in both monolingual and bilingual poststroke populations (Billot et al., 2022; Grasemann et al., 2021).

In clinical practice, ML and AI approaches could be used in the future to augment—not supplant—clinical decision making and reduce clinician workload. Liss and Berisha (2020) recently highlighted four areas of clinical practice likely to be positively affected by advancements in algorithmic technologies: (a) more efficient documentation practices, (b) better assistive technology for patients, (c) strides in objective assessment, and (d) greater degree of personalized practice. The potential in ML for a greater degree of personalized or precision medicine has likewise been forecasted by other researchers. In this primer, we have highlighted, for instance, research using these approaches for more accurate individual-level prediction of therapy-induced language recovery (Billot et al., 2022; Grasemann et al., 2021). AI approaches are being embedded as part of commercial therapy apps, such as Constant Therapy, which uses a proprietary “NeuroPerformance Engine” algorithm to prescribe specific task types and difficulty levels based on an individual users’ recent performance (Constant Therapy Health, n.d.; Dadgar-Kiani & Anantha, 2020). Algorithmic approaches are also the subject of ongoing grant-funded research for improved speech therapy approaches, such as the ChainingAI speech therapy software that uses ML models to learn “correct” and “incorrect” target sound productions and give tailored user feedback accordingly (Speech Motor Chaining, n.d.).

Despite the potential promise of ML and related technology, there remain significant barriers to its adoption by a clinical audience at the current moment in time. In this primer, we have discussed some of the more fundamental of these barriers—including issues of terminology, reliability, validity, bias, and interpretability—as well as potential solutions. A thorough accounting of these barriers is a necessary first step toward the development of the next generation of ML approaches that are reliable, clinically validated, and meaningful to clinician and patient end-users.

## Figures and Tables

**Table 1.** Key machine learning (ML) terminology.

ML term	Definition	Example
Algorithm	A sequence of rules executed by a computer to solve a problem	A one-layer decision tree can be represented as: For each patient, if their Western Aphasia Battery Aphasia Quotient is greater than 93.7, they are classified as within normal limits; else they are classified as having aphasia.
Data set	A collection of data from which ML models learn	An example data set contains multiple patients; for each patient, the data set contains information about aphasia severity, structural connectivity, functional connectivity, anatomical integrity, and whether or not the patient responds to speech-language therapy (SLT).
Model	A program that can find patterns or make decisions from previously unseen data. It represents what algorithms learned from training data.	After the training process, we can input data of a new patient to the model, including aphasia severity, structural connectivity, functional connectivity, and anatomical integrity. The model will predict whether or not the patient responds to SLT.
Example/sample	A single instance of combination of features and label	In a data set containing multiple patients, each patient is an example.
Feature	An independent property used as input to the model	In the previous example, aphasia severity, structural connectivity, functional connectivity, anatomical integrity are four features; the model predicts target value based on these input features.
Feature set	A collection of all features used in a model	In the previous example, the combination of four features is called the feature set.
Label/target	The predetermined outcome to be predicted by the model	In the previous example, whether the patient responds to SLT (i.e., responder vs. nonresponder) is the target value. Target could also be continuous, e.g., a test score.
Hyperparameter	Preset values as configuration settings to define the structure of a model	For a neural network, the number of layers and number of neurons in each layer are two important hyperparameters defined before the training process.
Training	The process whereby the model learns the hidden pattern and relationship existing in the features and how the features are related to target value	In the training process, a part of the data set is fed to the model, and the model can learn which features make significant contributions to prediction and which features are less important.
Validation	The process of selecting the best model and/or best configuration from many options by evaluating model performance on previously unseen data	After the training process, another part of the data set is used to evaluate the performance of the model. Training process and validation process will be executed multiple times to evaluate various kinds of algorithms, and multiple hyperparameter settings of each algorithm, to decide the best one.
Testing	The process of evaluating final performance of a model with optimal configuration on some other, previously unseen data	After the best model and hyperparameter(s) are selected, another part of the data set different from training and validation data set is used to examine the final performance of selected model.
Parameter/weight	A variable that is internal to the model and learned from training data	In the decision tree example (see *algorithm* term), the value of *k* is a parameter, and the algorithm will learn the optimal value of *k* in the training process.
Loss function	A function to measure the error between true labels and predictions. The goal of the training process is to minimize loss.	One simple loss function for classification is add 1 to total loss for each misclassified sample in the data set and minimize it.

**Table 2.** Commonly used evaluation metrics for different types of machine learning tasks.

Metric	Task	Definition
Mean square error (MSE)	Regression	The average of squares of errors. Here, error represents the difference between predict target value and true value.
Mean absolute error	Regression	Similar to MSE but take the absolute value of error instead of square.
*R*^2^ (coefficient of determination)	Regression	The proportion of the variation in the target value that is predictable from the features.
Accuracy	Classification	The proportion of correctly predicted results among all examples.
Precision	Classification	The proportion of true positive results among all predicted positive examples.
Recall	Classification	The proportion of true positive results among all actual positive examples.
F1	Classification	The harmonic mean of precision and recall, balancing the two values.
Silhouette coefficient	Clustering	Measures the mean distance between a sample and all other points in the same class as well as mean distance between a sample and all other points in the nearest cluster. Higher values indicate that the example is well matched in current cluster.

*Note*. F1 = harmonic mean of precision and recall.

**Table 3.** Exemplar aphasiology studies using machine learning (ML) approaches for subgroup diagnosis and/or severity characterization.

Study	Population (*N*)	Predicted outcome(s)	Predictor variables^a^	Variable preselection?	ML approach(es)	Classification vs. regression	Evaluation metric(s) reported	Feature ranking/interpretation?
Pustina et al. (2017)	Chronic poststroke aphasia (53)	WAB AQ WAB Repetition WAB Auditory Comprehension PNT # correct	Lesion maps Structural connectivity Functional connectivity	Yes, recursive feature elimination	RF w/ stacked multimodal prediction	Regression	Pearson’s *r*; RMSE	Yes, top 1 feature per outcome
Kristinsson, Zhang et al. (2021)	Chronic poststroke aphasia (116)	WAB AQ WAB Fluency WAB Spontaneous Speech WAB Naming WAB Repetition WAB Auditory Comprehension	Cerebral blood flow Lesion load Fractional anisotropy fMRI	Yes, univariate regression analysis (per outcome variable)	Support vector regression	Regression	Pearson’s *r*; MSE	Yes, comparative prediction accuracy by predictor variable modality per outcome
Themistocleous et al. (2021)	Primary progressive aphasia (44)	Subgroup diagnosis: nfvPPA lvPPA svPPA	Acoustic predictors Morphosyntactic predictors	No	DNN SVM DT RF	Classification (supervised)	Accuracy; precision; recall; F1	No
Fromm et al. (2022)	Poststroke aphasia (168)	Subgroup diagnosis (unsupervised^a^)	Connected speech predictors (lexical, syntactic, etc.) Demographics Standardized aphasia test scores	No	*K*-means clustering RF (nested subanalysis)	Classification (unsupervised)	Proportion agreement (*k*-means vs. RF groupings); confusion matrices	Yes, average variable importance (RF subanalysis)

*Note*. WAB AQ = Western Aphasia Battery–Revised Aphasia Quotient (Kertesz, 2006); PNT = Philadelphia Naming Test; RMSE = root-mean-square error; fMRI = functional magnetic resonance imaging; MSE = mean square error; nfvPPA = nonfluent variant primary progressive aphasia; lvPPA = logopenic variant primary progressive aphasia; svPPA = semantic variant primary progressive aphasia; DNN = deep neural network; SVM = support vector machine; DT = decision tree; RF = random forest; F1 = harmonic mean of precision and recall.

a Primary analysis approach in this study was an unsupervised *k*-means clustering analysis; supporting supervised RF analysis was also run.

**Table 4.** Exemplar aphasiology studies using machine learning (ML) approaches for prediction of recovery.

Study	Population (*N*)	Predicted outcome(s)	Predictor variables	Variable preselection?	ML approach(es)	Classification vs. regression	Evaluation metric(s) reported	Feature ranking/interpretation?
Billot et al. (2022)	Chronic poststroke aphasia (55)	Responder vs. nonresponder to SLT	Structural connectivity Functional connectivity Demographics Behavioral performance Anatomical integrity	Yes, supervised feature selection	SVM RF	Regression	Accuracy, F1, precision, recall	Yes, model runs per feature/feature combination
Grasemann et al. (2021)	Chronic bilingual (Spanish–English) poststroke aphasia (13)	Pre- and poststroke naming ability Tx response in treated and untreated language	Demographics Pre- and poststroke bilingual language background Behavioral performance	Yes, domain knowledge-based feature selection	Neural network model based on self-organizing maps	Regression	Correlation (*R*^2^)	No

*Note*. SLT = speech-language therapy; SVM = support vector machine; RF = random forest; F1 = harmonic mean of precision and recall; Tx = treatment.

## Data Availability

No primary data were analyzed for the current tutorial.
